# COVID-19–associated lncRNAs as predictors of survival in uterine corpus endometrial carcinoma: A prognostic model

**DOI:** 10.3389/fgene.2022.986453

**Published:** 2022-09-06

**Authors:** Yang Ding, Xia Li, Jiena Li, Liqun Zhu

**Affiliations:** ^1^ Department of Obstetrics and Gynaecology, The Chinese University of Hong Kong, Shatin, HongKong, China; ^2^ Department of Obstetrics and Gynaecology, Heze Municipal Hospital, Heze, Shandong, China; ^3^ Department of Emergency Medicine, The First Affiliated Hospital of Harbin Medical University, Harbin, Heilongjiang, China

**Keywords:** endometrial carcinoma, COVID-19–associated lncRNA, TCGA, OS, prognostic model

## Abstract

**Background:** Patients with uterine corpus endometrial carcinoma (UCEC) may be susceptible to the coronavirus disease-2019 (COVID-19). Long non–coding RNAs take on a critical significance in UCEC occurrence, development, and prognosis. Accordingly, this study aimed to develop a novel model related to COVID-19–related lncRNAs for optimizing the prognosis of endometrial carcinoma.

**Methods:** The samples of endometrial carcinoma patients and the relevant clinical data were acquired in the Carcinoma Genome Atlas (TCGA) database. COVID-19–related lncRNAs were analyzed and obtained by coexpression. Univariate, least absolute shrinkage and selection operator (LASSO), and multivariate Cox regression analyses were performed to establish a COVID-19–related lncRNA risk model. Kaplan–Meier analysis, principal component analysis (PCA), and functional enrichment annotation were used to analyze the risk model. Finally, the potential immunotherapeutic signatures and drug sensitivity prediction targeting this model were also discussed.

**Results:** The risk model comprising 10 COVID-19–associated lncRNAs was identified as a predictive ability for overall survival (OS) in UCEC patients. PCA analysis confirmed a reliable clustering ability of the risk model. By regrouping the patients with this model, different clinic-pathological characteristics, immunotherapeutic response, and chemotherapeutics sensitivity were also observed in different groups.

**Conclusion:** This risk model was developed based on COVID-19–associated lncRNAs which would be conducive to the precise treatment of patients with UCEC.

## Introduction

Uterine corpus endometrial carcinoma (UCEC) has been found as the second incident carcinoma and the leading cause of death among female carcinoma patients for its high recurrence rates (Shen et al., 2018; Zhang et al., 2019). It is an endometrial epithelial malignancy with a high mortality rate and a severe threat to women’s health (Chen et al., 2016; Morice et al., 2016). Non-estrogen–dependent tumors have a lower incidence, but are highly malignant and have a poor prognosis (Suarez et al., 2017; Tang et al., 2019). In recent years, the survival rate of patients with advanced UCEC and metastatic endometrial carcinoma has decreased significantly (Carlson and Nastic, 2019). In patients with metastases, conventional first-line treatment is ineffective and can cause more severe damage to normal cells. Increasing studies have shown that some patients could show contrasting clinical outcomes and characteristics within the identical stage group. Thus, there is an urgent need for more effective and accurate methods to predict the survival of patients with UCEC (Geng et al., 2020). As a new pillar of modern carcinoma treatment, immuno-oncology is revolutionizing carcinoma therapy (Miller et al., 2019).

Tumor mutation burden (TMB) is a novel biomarker that refers to the total number of mutations in tumor tissue (Yang et al., 2022). A large amount of evidence indicates that the tumor mutation burden is related to immunotherapy in carcinoma patients, so it is necessary to study further the immune invasiveness of TMB on UCEC (Jones et al., 2020). Therefore, we focused on investigating the clinical prognosis of UCEC related to TMB and immune function.

Coronavirus disease-2019 (COVID-19) has been detected in Wuhan, Hubei Province, China since December 2019 (Zhang et al., 2021). Carcinoma patients have been reported to be at higher risk of severe events with COVID-19, mainly due to systemic immunosuppression caused by malignancy and anticarcinoma treatments such as chemotherapy or surgery (Longbottom et al., 2016). The poorer prognosis for carcinoma patients from COVID-19 is a timely reminder that we should take carcinoma patients more seriously (Zhou et al., 2020). However, the prognosis of COVID-19 patients with UCEC is still unclear. Our research focuses on the immune molecular characteristics of UCEC and immunotherapeutic intervention. With the in-depth study of lncRNAs and the immune system, scholars have realized that COVID-19–related lncRNAs are potential prognostic biomarkers and may provide new treatment options (Ma et al., 2020; Yang et al., 2020). However, we used Cox and lasso regression analysis to identify a COVID-19–related lncRNA molecular feature for UCEC patients.

Here, we analyzed TCGA dataset of lncRNA expression in UCEC and screened for lncRNA markers related to COVID-19. Our results validate a risk scoring model for 10 COVID-19 immunization–associated lncRNAs. The model can be used as a reliable prognostic predictor and the 10 lncRNAs can be used as potential therapeutic targets for endometrial carcinoma.

## Methods and methods

### Data source

The transcriptome profiles were obtained, and clinical information of the UCEC cohort was extracted separately from TCGA database. Patients with missing OS data were excluded from the statistical study to avoid further biases. Last, we acquired entire TCGA dataset consisting of 541 patients (Li et al., 2020a).

### Selection of COVID-19 genes and COVID-19–associated lncRNAs

Genes related to COVID-19 were selected from the GeneCards database, OMIM database, and NCBI gene function module (Li et al., 2021). Pearson correlation analysis was performed to filter COVID-19–associated lncRNAs. Based on the criteria of Pearson |R| > 0.3 and *p* < 0.001, 1263 COVID-19–associated lncRNAs were identified (Li et al., 2020b; Deng et al., 2020; Hu et al., 2020).

### The construction and verification of the risk score

Entire TCGA was divided into training and testing sets. Where the training set was built, a COVID-19–associated lncRNA model and the testing set and the whole set were available to ensure the model’s performance. Combined with the UCEC survival information in TCGA, 40 COVID-19–associated lncRNAs were filtered out by performing the univariate Cox regression (*p* < 0.05). The application of multi-factor COX regression was then used to analyze the 18 COVID-19–associated lncRNAs, and a 10 COVID-19–associated lncRNA risk model was ultimately established. The subsequent formula was applied to compute the risk score as the previous research (Xu et al., 2020a). The UCEC patients were segmented into low-risk or high-risk groups based on average risk scores (Xu et al., 2020b).

### Principal component and Kaplan–Meier survival analyses

Principal component analysis (PCA) was used for effective dimensionality reduction, model identification, and grouping visualization of high-dimensional data of the entire gene expression profiles, COVID-19 genes, COVID-19–associated lncRNAs, and risk model according to the expression patterns of the 10 COVID-19–associated lncRNAs. The patients’ survival analysis adopted log-rank tests and Kaplan–Meier curves to analyze statistical differences between low- and high-risk score groups (Zhao et al., 2020).

### Chemotherapeutic and immunotherapeutic response predictions

We projected the 50% maximum inhibitory concentration (IC50) of chemotherapeutic agents in UCEC with the R package pRRophetic. The aforementioned relevant information was collected at the GDSC (Lu et al., 2019). TMB was defined as mutations per megabase in the genome and is an emerging therapeutic measure of sensitivity to immunotherapy (Chalmers et al., 2017). The TMB score of the respective patient with UCEC was obtained and divided by the size of the coding region of the target region.

## Results

### Data source and processing

We obtained 13,413 lncRNA expression and 339 COVID-19 gene expression profiles from UCEC RNA sequencing data in TCGA. By coexpression analysis of the immune gene list, we obtained 1,262 COVID-19 immunization–associated lncRNAs (|Pearson R|>0.3, *p* < 0.001), (Supplemental Figure S1). The entire group was then used to observe the clinical profile, immunological profile, and chemotherapy outcomes of patients in the high-risk and low-risk groups.

### Identification and validation of the COVID-19–associated lncRNA model in uterine corpus endometrial carcinoma patients

**FIGURE 1:**
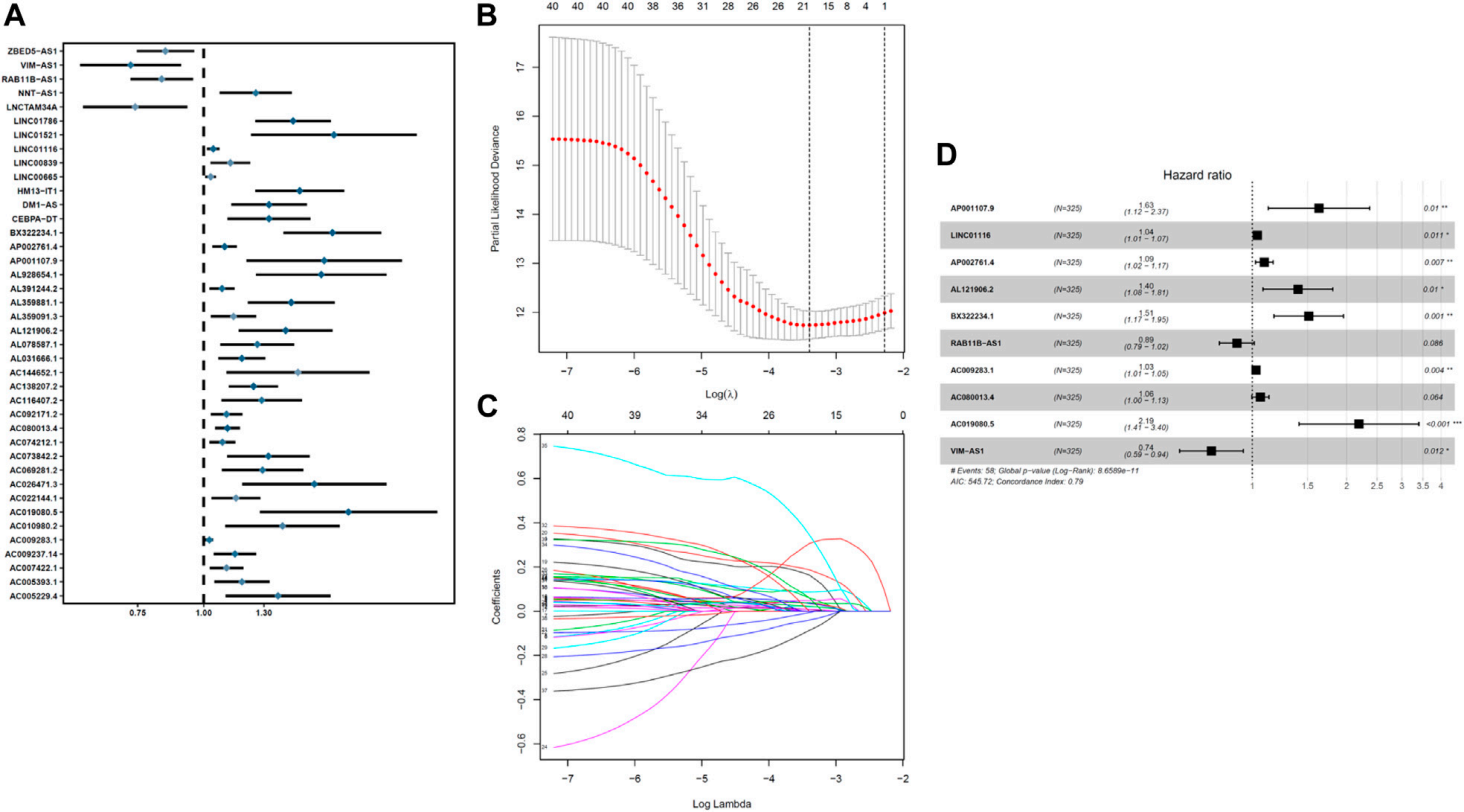
Identification and construction of a prognostic model for COVID-19–associated lncRNAs in UCEC patients. **(A)** Univariate Cox regression analysis revealed that the selected lncRNAs are significantly correlated with clinical prognosis. **(B–C)** Risk score system was constructed using the LASSO Cox regression model. **(D)** Multivariate Cox regression analysis shows independent prognostic lncRNAs.

The entire TCGA data were randomly assigned to the training and experimental groups, of which 325 patient samples were assigned to the training group and 216 patient samples were allocated to the experimental group. We performed a univariate Cox regression analysis of the expression profiles of COVID-19–associated lncRNAs in the training set and obtained 40 lncRNAs that were significantly related to OS, (*p* < 0.01) (Figure 1A). We performed LASSO-penalized Cox analysis to select 18 lncRNAs related to COVID-19 that were significantly related to OS (Figures 1B,C). Ten COVID-19 immunization–associated lncRNAs were obtained, namely, AP001107.9, LINC01116, AP002761.4, AL121906.2, BX322234.1, RAB11B-AS1, AC009283.1, AC080013.4, AC019080.5, and VIM-ASI (Figure 1D). The expressions of all the 10 COVID-19 immune–associated lncRNAs in UCEC are presented in Figure 1D.

**FIGURE 2:**
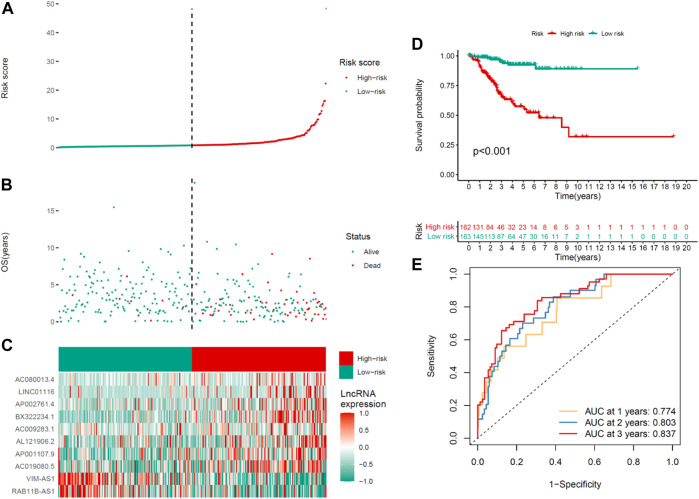
Prognosis ability of the risk model of the 10 COVID-19–associated lncRNAs in the training set. **(A)** Distribution of COVID-19–associated lncRNA model–based risk score. **(B)** Situations of the survival period and survival state between high- and low-risk groups. **(C)** Clustering analysis heatmap shows the expression levels of the 10 prognostic lncRNAs for the respective patient. **(D)** Kaplan–Meier survival curves of OS of patients in the high- and low-risk groups. **(E)** 1-, 2-, and 3-year ROC curves for OS prediction in accordance with COVID-19–associated lncRNAs.

The average risk score is a non-negligible basis for classifying low- and high-risk groups in UCEC samples. The general condition of risk scores in the two groups were described in Figures 2A,B showed the distinctions between the survival state and survival period of the low-risk group and the high-risk group. The associated expression situations of the 10 COVID-19–associated lncRNAs per patient are illustrated in Figure 2C.

Significantly, the OS of the lower-risk group was shorter than that of the high-risk group in the Kaplan–Meier survival analysis (*p* < 0.001) (Figure 2D). The ROC curves revealed that the COVID-19–associated lncRNA model exhibited a potentiality to forecast OS (1-year AUC = 0.774, 2-year AUC = 0.803, and 3-year OS = 0.837; Figure 2E).

**FIGURE 3:**
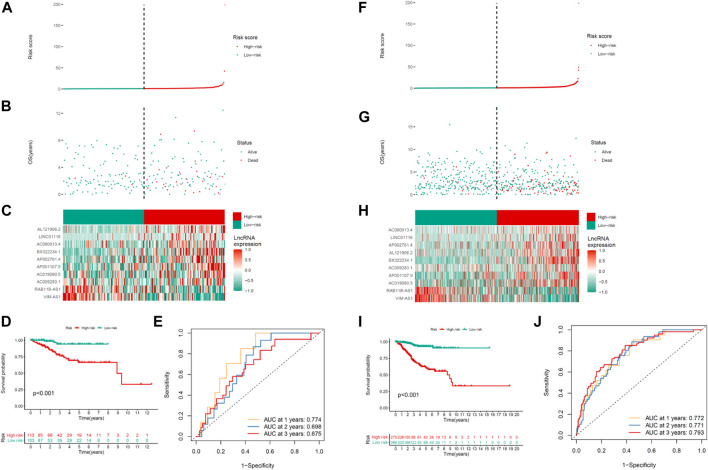
Prognosis ability of the risk model of the 10 COVID-19–associated lncRNAs in the testing and whole sets. Distribution of the risk score, OS, gene expression, survival analysis, and ROC curves for forecasting OS in the **(A–E)** testing set and **(F–J)** entire set.

Proving the predictability of COVID-19–associated lncRNA model, the risk scores for the respective patient were obtained in the testing and the whole set used the same formula. Figure 3 depicts the distribution of risk scores, the survival period and survival state, and the expression of the COVID-19–associated lncRNAs in the testing set (Figures 3A–C) and the whole set (Figures 3F–H). This study had higher OS in the UCEC high-risk group, which is consistent with the training set results. Kaplan–Meier survival analysis carried out the testing and the whole set coincided with the outcomes in the TCGA training set: patients with the UCEC with lower-risk scores had longer OS (Figures 3D,I). The ROC analysis illustrated that the COVID-19–associated lncRNA model had accurate predictability on UCEC in the testing set (1-year AUC = 0.774, 2-year AUC = 0.698, and 3-year AUC = 0.675; Figure 3E) and the whole set (1-year AUC = 0.772, 2-year AUC = 0.771, and 3-year AUC = 0.793; Figure 3J).

**FIGURE 4:**
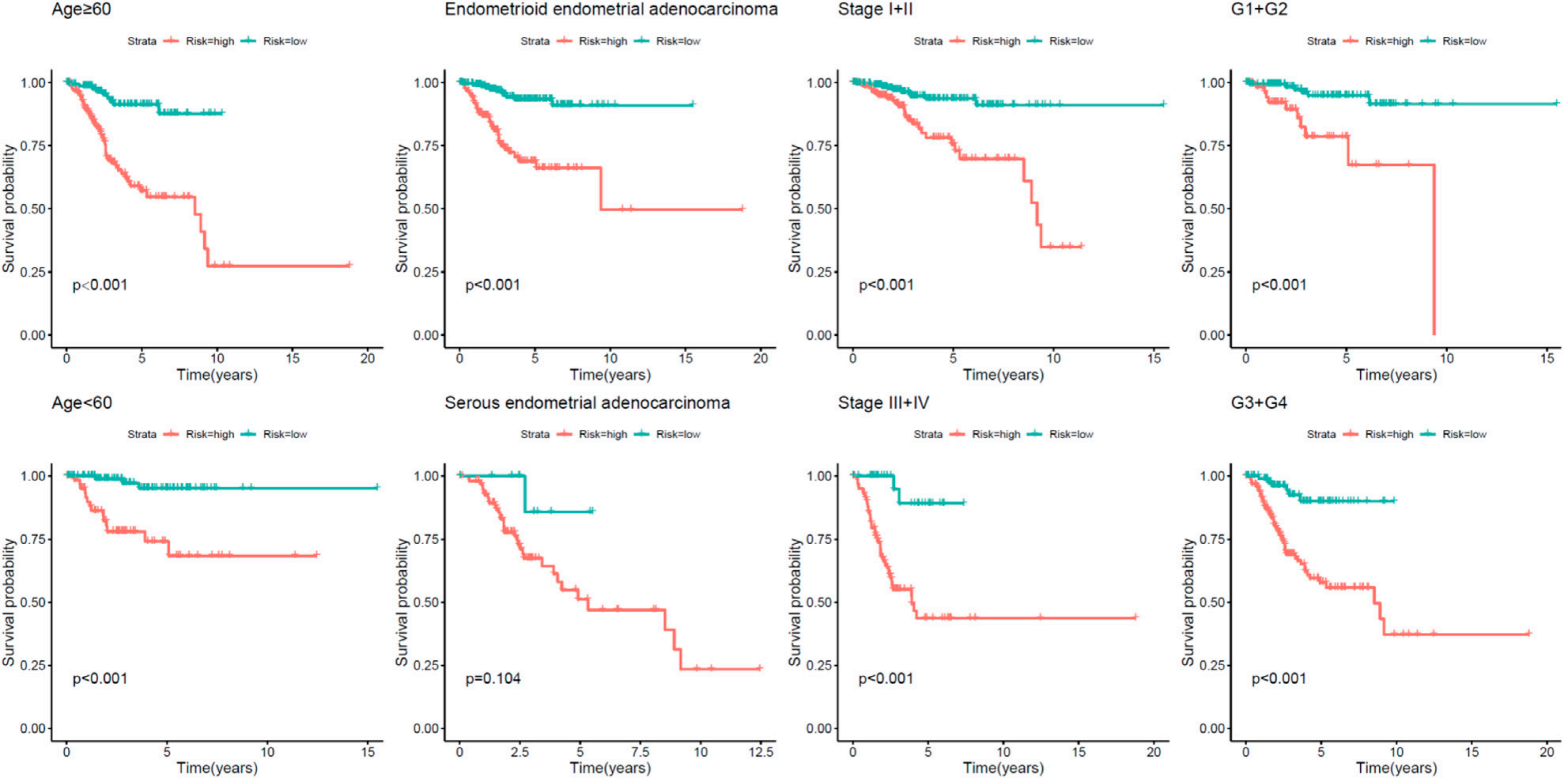
Kaplan–Meier analysis of overall survival for UCEC patients according to age, event, histological type, and tumor stage.

Afterward, stratified by the universal clinicopathological parameters, the discrepancies in OS between the two risk groups were analyzed in the TCGA whole set. Just as depicted in Figure 4, the low-risk group had continuously better OS than the high-risk group when classified by age, histological type, and tumor stages.

### Principal component analysis further verifies the grouping ability of the COVID-19–associated lncRNA model

**FIGURE 5:**
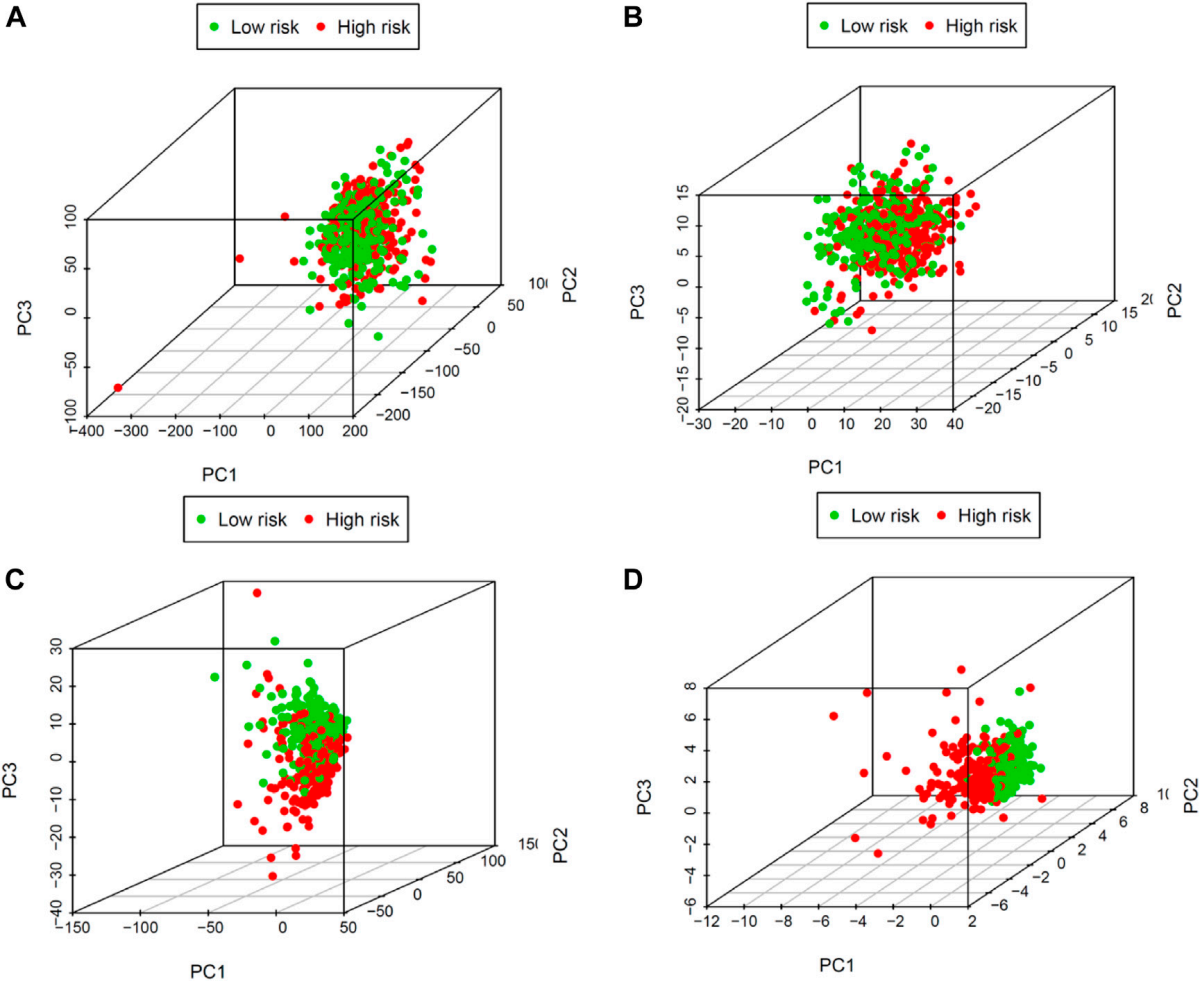
Principal components analysis between low- and high-risk groups based on **(A)** the entire gene expression, **(B)** COVID-19 genes, **(C)** COVID-19–associated lncRNAs, and **(D)** the risk model.

PCA examines differences between low- and high-risk groups based on the entire gene expression (Figure 5A), COVID-19 genes (Figure 5B), COVID-19–associated lncRNA genes (Figure 5C), and the risk model (Figure 5D). Our model results manifested that the low- and high-risk groups are generally distributed in different directions. Nevertheless, the distribution of the high-risk and low-risk groups shown in Figures 5C,D is relatively dispersed, which confirms that our prognostic characteristics were able to distinguish between the high- and low-risk groups.

### Clinical evaluation by the risk assessment model

**FIGURE 6:**
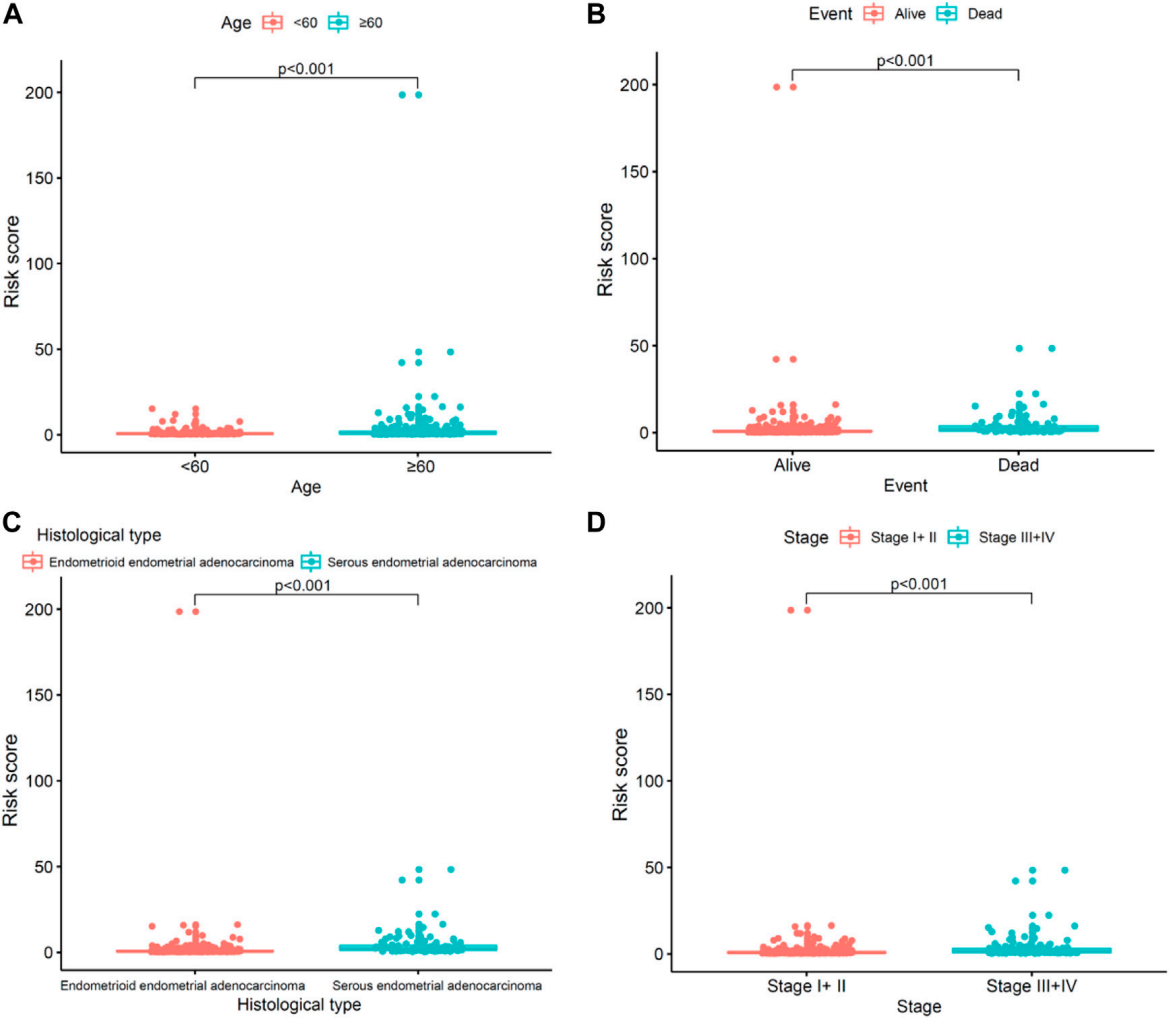
Clinical evaluation by the risk assessment model. **(A–D)** Scatter diagram shows the **(A)** age, **(B)** event, **(C)** histological type, and **(D)** clinical stage.

We explored the relationship between the risk model and clinicopathological characteristics. Operated by the Wilcoxon signed-rank test, the strip chart and consequent scatter diagrams showed that age(Figure 6A), event (Figure 6B), histological type (Figure 6C), and survival state (Figure 6D) were obviously correlated to the risk. Univariate and multivariate Cox regression analyses were further utilized to probe whether the COVID-19–associated lncRNA model was independent of current clinical pathological prognostic indicators. To evaluate the sensitivity and specificity of the predictability of this risk score in the prognosis of patients with UCEC, the concordance index was obtained. The consistency index of the risk score outperformed other clinical factors over time, suggesting that the risk score can be beneficial to the prognosis of UCEC.

### Evaluating correlations of the COVID-19–associated lncRNA model with immunotherapeutics and chemotherapeutics in patients with uterine corpus endometrial carcinoma

**FIGURE 7:**
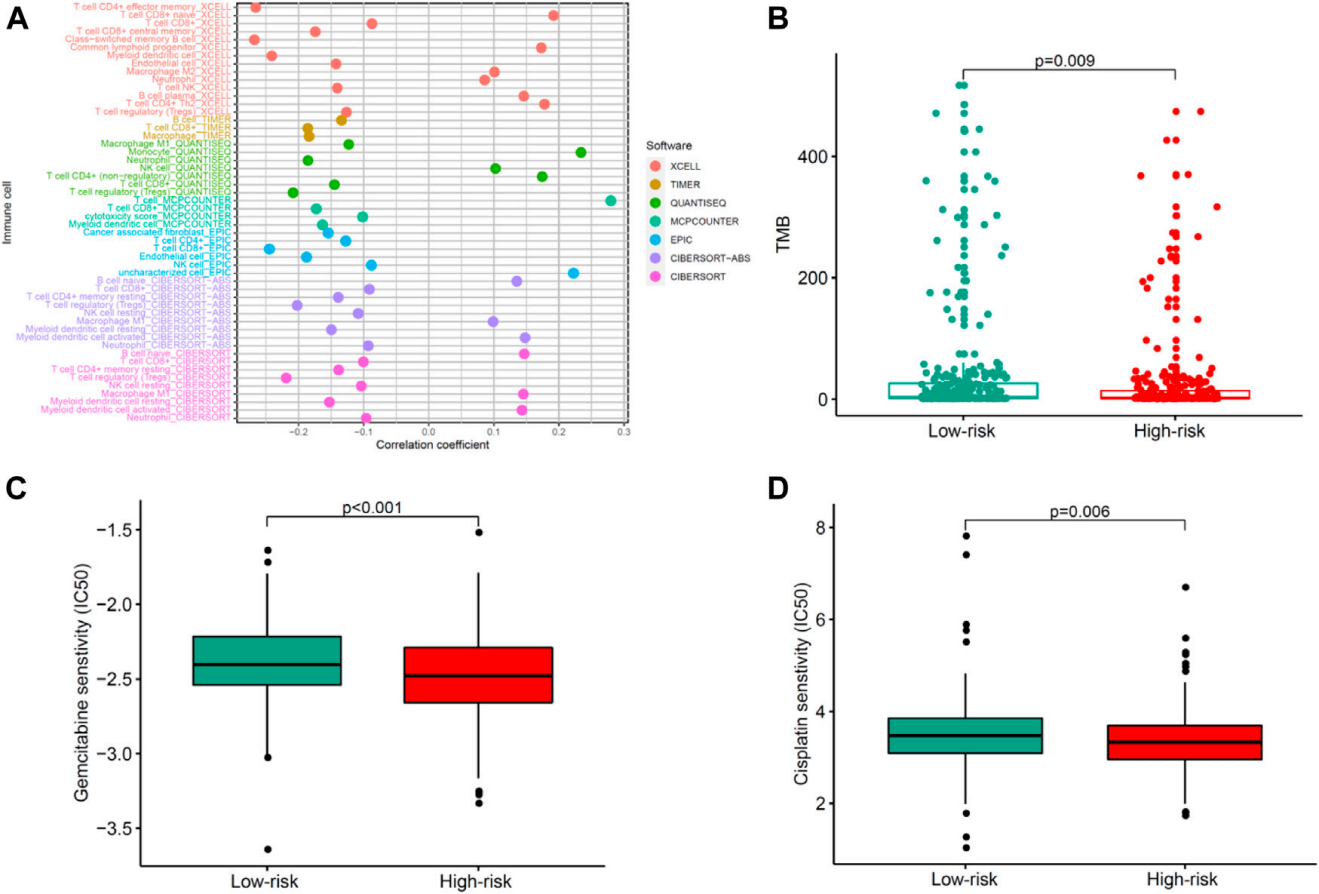
Evaluating correlations of the COVID-19–associated lncRNA model with immunotherapeutics and chemotherapeutics in patients with UCEC. **(A)** Tumor-infiltrating immune cells and **(B)** TMB differences in high- and low-risk groups. **(C–D)** Differential response of chemotherapeutics such as **(C)** gemcitabine and **(D)** cisplatin based on IC50 for UCEC patients in high- and low-risk groups.

To explore the relationship between the risk model and tumor-infiltrating immune cells, we used seven standard acceptable methods to estimate the immune infiltrating cell (Hong et al., 2020). The result showed that the risk model was related to the tumor immune micro-environment (Figure 7A). To explore the immunotherapeutic response, we detected the expression of TMB. As presented in Figure 7B, high-risk patients had a lower expression of TMB than low-risk patients, indicating that immunotherapy may be beneficial for low-risk patients. Common chemotherapeutics (e.g., gemcitabine and cisplatin) were adopted to identify the potential clinical efficacy in treating patients with UCEC. The result showed that low-risk was associated with a higher half inhibitory centration (IC50) of chemotherapeutics such as gemcitabine and cisplatin, which indicated that the model acted as a potential predictor for chemosensitivity (Figures 7C,D). Collectively, the aforementioned data highlight the potential clinical significance of the proposed model in the identification of immunotherapeutics and chemotherapeutics in patients with UCEC.

## Discussion

UCEC has been found as one of the most ordinary types of gynecologic malignancy worldwide with rising incidence and mortality rates (Wang et al., 2020). Currently, cancer treatment methods for UCEC include surgery, chemotherapy, and radiotherapy (Liu et al., 2020). These treatments can cure cancer in the early stage, but they are often ineffective for cancer in the late or recurrent stage. Immunotherapy is an effective treatment for cancer, mainly for the clinical treatment of recurrent or metastatic tumors (Igarashi and Sasada, 2020; Quaglino et al., 2020). Basic and clinical studies have shown that antitumor immunity plays a key role in the development and progression of cancer (Cha et al., 2020). Patients with UCEC reflect resemble clinical features, but due to molecular heterogeneity, they have distinct clinical results (Li et al., 2020c). Recently, a few research studies have reported that long non–coding RNAs (lncRNAs) as biomarkers have a promising ability for diagnosis and prognostics in different types of UCEC (Sun et al., 2017; Ye et al., 2020). Moreover, lncRNAs have highlighted the vital roles in tumorigenesis and development (Gutschner and Diederichs, 2012; Zhou et al., 2018). Nevertheless, the function of COVID-19–associated lncRNAs in UCEC remains indistinct (Piergentili et al., 2021).

COVID-19 is an emerging and rapidly evolving epidemic disease (Severe Outcomes Among Patients with, 2020), and specific treatments for 2019 coronavirus disease have not yet been developed for the current global infection rate. Meanwhile, the incidence of malignant diseases such as carcinoma has increased rapidly in recent decades (Chandy et al., 2020). Patients with carcinoma have weakened immune systems, develop immunosuppression and immune dysfunction, and any external infection can worsen their health (Liu et al., 2020; Rahman et al., 2021). However, the risk profile, prognosis, and treatment outcome of carcinoma patients remain unclear. There are few studies on the mechanism of COVID-19–associated lncRNAs in UCEC currently. Some research studies about identifying reliable biomarkers for UCEC prognosis prediction have been carried out. According to the current literature, we analyzed the incidence, clinical features, and treatment outcome of UCEC patients infected with COVID-19. Patients with UCEC are susceptible to COVID-19 and the prevalence of the disease in patients with UCEC may worsen treatment. This study aimed to deepen research to gain more insights into the biological effect and prognosis of COVID-19 infection in patients with UCEC and then improve the clinical management of the earlier patients.

Even to this day, traditional clinicopathological features are still the most crucial contributing factors affecting the diagnosis and treatment of UCEC (Liu and Jing, 2022). The existing clinical characteristics are insufficient to accurately predict the prognosis, probably to the extent that there are differences in the prognosis of patients with similar clinical features (Li and Wan, 2020). In the current study, the lack of a validation cohort limits the prognostic value of biomarkers. We used Cox and lasso regression analyses to identify a new immune-related lncRNA molecular feature. Then, UCEC patients were effectively divided into the high-risk group and low-risk group in the test group and the entire group. Compared with the high-risk group, the overall survival time of the low-risk group was significantly longer. In addition, the results showed good predictive performance and proved to be repeatable and reliable for cancer prognosis. In this study, our model was a robust independent prognostic factor in OS prediction with high accuracy. Importantly, a concordance index was established, combining the risk model with UCEC clinical data, to predict OS, implying that COVID-19–associated lncRNAs as a signature was better at predicting the 1‐, 2‐and 3‐year OS of patients with UCEC.

In this study, we established an immune prognostic feature to observe the immune status of UCEC patients and predict clinical results. Our data show a significant correlation between prognostic characteristics and tumor grade in terms of the clinical effect (*p* < 0.001), which means that the risk score calculated by our model is significantly higher in advanced cases. In addition, when patients are stratified by age, the constructed prognosis model also shows the potential to predict the differential prognosis between high-risk and low-risk groups (<60 and ≥60), histological type (endometrioid endometrial adenocarcinoma and serous endometrial adenocarcinoma), clinical stages (I + II and III + IV), and event (alive and dead). This shows that this study is effective in predicting the prognosis of patients under different clinical and pathological conditions. Next, PCA confirmed that our prognostic characteristics have good grouping ability. The model constructed in this study has played its advantages in preliminarily determining the prognosis of patients and adjusting the treatment plan according to the expression of immune genes and the level of immune cell infiltration.

The current study adopts a new prognostic method for immune-related endometrial cancer. We performed more functional studies on 10 COVID-19–associated lncRNAs to further discover potential immune-related mechanisms. It is worth noting that we are currently conducting clinical validation and mechanism interpretation of these results. In various studies, gene expression differences between cancer and normal tissues were compared to screen prognostic genes. This may omit genes with slight differences, but these genes with insignificant differences may have a great impact on the biological behavior of tumors, chemotherapy, immunotherapy, and other factors affecting the survival of patients. This study is the first to report external validation of the established COVID-19–associated lncRNA characteristics of endometrial cancer. However, 10 COVID-19–related lncRNAs have never been studied in UCEC patients. However, we also recognized that there were still some deficiencies and limitations in the research. First, retrospective data were used, and some heterogeneity may occur among patients with UCEC. Investigators designing more prospective cohort studies could validate the prognostic value of this risk model in a broader population. Second, further extensive molecular experiments are needed to reveal the potential mechanism of COVID-19–associated lncRNAs.

Conclusively, this research provides a promising avenue for facilitating the individualized survival prediction in patients with UCEC and may clarify the mechanism and process of lncRNA COVID-19 modification. Furthermore, the predictive model is conducive to screening clinical adaptation in patients with UCEC who respond well to chemotherapy and immunotherapy.

## Data Availability

The original contributions presented in the study are included in the article/Supplementary Material; further inquiries can be directed to the corresponding authors.
