# Supplementary figures and images for: COVID-19–associated lncRNAs as predictors of survival in uterine corpus endometrial carcinoma: A prognostic model
Source: Front Genet. 2022 Sep 6;13:986453. doi: 10.3389/fgene.2022.986453 (PMC9486303; doi:10.3389/fgene.2022.986453)

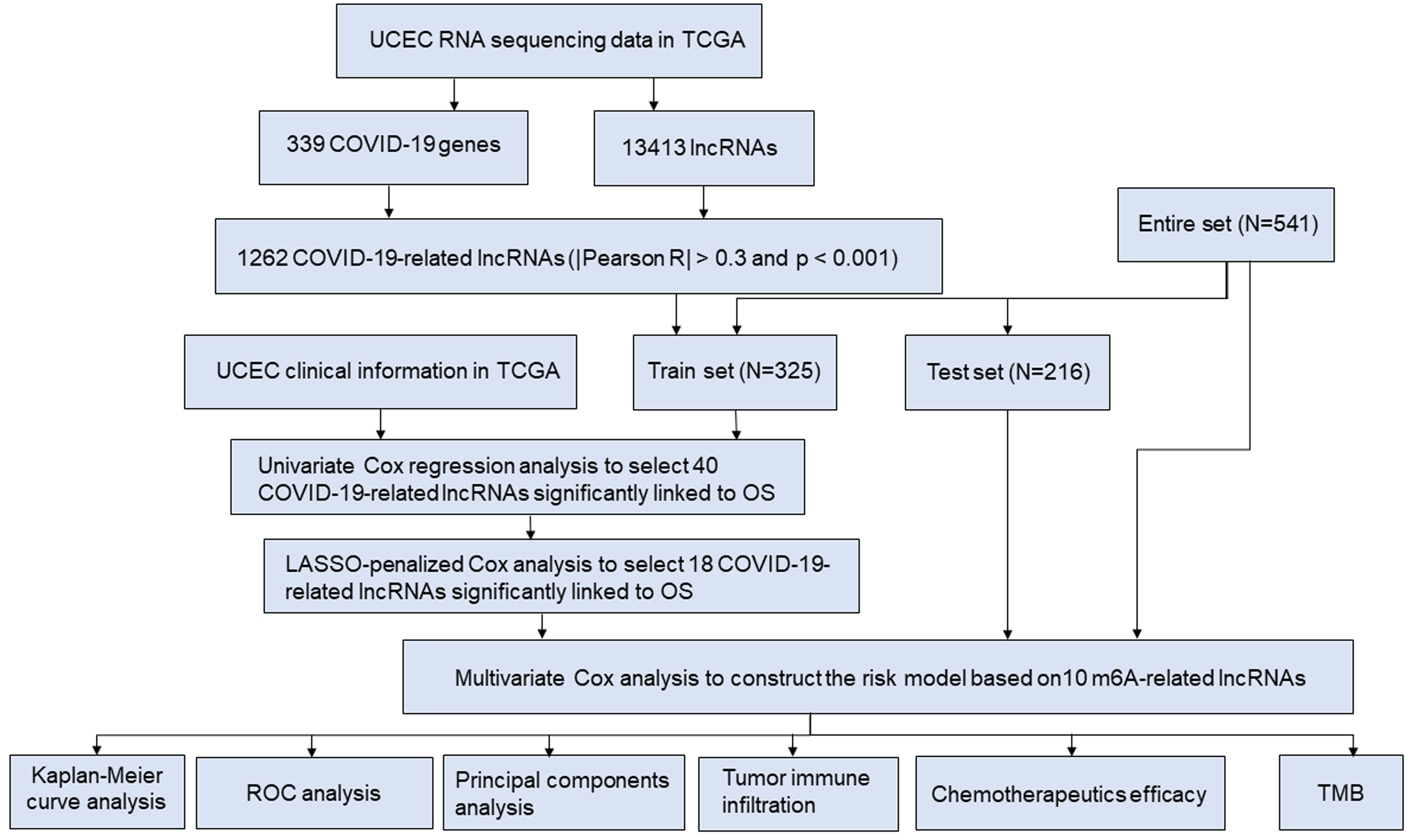

Supplement: Supplementary file 1 [file Image1.TIF]
